# Poverty and the health of children and adolescents at the end of the COVID-19 pandemic. Results of the KIDA study

**DOI:** 10.25646/13185

**Published:** 2025-06-25

**Authors:** Miriam Blume, Elvira Mauz, Mira Tschorn, Kristin Manz, Anja Schienkiewitz, Jennifer Allen, Jens Hoebel, Petra Rattay

**Affiliations:** 1 Robert Koch Institute, Department of Epidemiology and Health Monitoring, Berlin, Germany; 2 University of Potsdam, Social and Preventive Medicine, Potsdam, Germany

**Keywords:** Children, Adolescents, Poverty, Income, Health, School, Health behaviour, Health inequality, Pandemic, COVID-19

## Abstract

**Background:**

During the COVID-19 pandemic, young people at risk of poverty were particularly affected by contact restrictions as well as by daycare centre and school closures. The aim here is to describe the health status of young people at risk of poverty in comparison to their peers from financially better-off families at the end of the pandemic.

**Methods:**

The analyses are based on the data of 3- to 15-year-olds from the study German Children’s Health Update (2022/2023). Prevalences stratified by income were determined for selected indicators of health, health-related behaviour and psychosocial stress and resources. A comparison was made between families at risk of poverty and families with medium and high incomes. Poisson regressions were adjusted for parents’ level of education.

**Results:**

Young people at risk of poverty are more likely to have poor health than their peers from financially better-off families. While the former are less likely to participate in organised sport in their leisure time, there are no differences in participation in voluntary sports activities at school according to family income.

**Conclusions:**

Strategies to reduce health-related disadvantages for young people at risk of poverty must be implemented at the level of society as a whole and in local settings. Continuous monitoring of children’s and adolescents’ health can help to identify trends at an early stage.

## 1. Introduction

According to Article 24 of the UN Convention on the Rights of the Child, all children have the right to the highest attainable standard of health [1]. However, the health opportunities of children and adolescents are unevenly distributed, even in a comparatively affluent country like Germany. Studies conducted for Germany show that children and adolescents who are at risk of poverty are more likely to have poorer health than their peers from financially better-off families [2–4].

According to the microcensus in 2022, 3.1 million children and adolescents under the age of 18 were at risk of poverty in Germany, more than one in five children (21.8 %) [5]. Children and adolescents from single-parent households [6], from families with many children [6] and from families with low education [7] or longer periods of unemployment [4] are particularly at risk of poverty. Poverty is characterised by numerous economic, social and cultural restrictions [8] and has an impact on almost all areas of children’s and adolescents’ lives. Children and adolescents at risk of poverty often experience deprivation, austerity, stigmatisation and shame in their lives [6]. Children and adolescents at risk of poverty often live in cramped living conditions with reduced opportunities to withdraw [3] and a less stimulating living environment [9]. In addition, families at risk of poverty often do not have enough money for a good quality, healthy diet [10]. Young people at risk of poverty also often experience disadvantages in the education system, which is reflected, for instance, in the strong correlation between family income and attendance at a ‘Gymnasium’ – the academically oriented secondary school track leading to university entrance qualifications [11]. Children and adolescents from families at risk of poverty therefore usually have poorer opportunities for development, education and good health from an early age [6].

The COVID-19 pandemic exacerbated the social disadvantage of poor children and adolescents in some respects [12], as young people from families at risk of poverty were particularly exposed to stress due to containment measures such as temporary contact restrictions, daycare and school closures or homeschooling [13]. Smaller homes and fewer financial resources made it difficult to provide care in the event of school closures and distance learning. In addition, parents with low levels of formal education were often unable to support children in homeschooling in the same way as parents with higher levels of formal education [13]. In the months at the end of and after the pandemic, rising prices for food, housing costs and mobility also had a greater negative impact on families living in poverty [1].

Internationally and for Germany, studies show that mental health problems [14–17] and lack of physical activity [18] increased in children and adolescents during the pandemic and have not returned to pre-pandemic levels afterwards [16].

Studies also show that socially disadvantaged children and adolescents in Germany had poorer health during and after the pandemic than their more affluent peers [12, 19–22]. The existing studies on health inequalities in children and adolescents in Germany during the COVID-19 pandemic focused on health differences and burdens depending on family wealth [19], multidimensional measures of social disadvantage [20, 21] or the socioeconomic situation of the living environment [12]. Differences depending on the family’s financial situation were only analysed in a survey of insured persons conducted by the Scientific Institute of the AOK at the beginning of 2022 [22]: both the physical and mental health of children was rated as very good or good more often by parents with a medium or high income than by parents with a low income. Children from low-income families were also affected by a lack of exercise more frequently than average. Although the study categorises family income, no data is reported explicitly for children and adolescents at risk of poverty. No nationwide results are currently available specifically on the health status of children and adolescents at risk of poverty in the context of the COVID-19 pandemic.

**Key messages**

► At the end of the pandemic, the health of children and adolescents at risk of poverty was worse than that of their peers from families with higher incomes.
► Children and adolescents at risk of poverty can be quite easily reached through programmes in the school setting: participation in voluntary sports activities at school was independent of income.
► Children and adolescents at risk of poverty are more burdened by financial restrictions and cramped living conditions than their peers from families with higher incomes.
► The utilisation of psychosocial support services with or for their children is higher among parents at risk of poverty than among families with higher incomes.
► Continuous monitoring is required to be able to analyse trends in health inequalities.

Taking this into account, the aim of this article is to provide an overview of the health status of children and adolescents from families at risk of poverty compared to financially better-off families at the end of the pandemic based on nationwide data.

To this purpose, the article examines the following questions:

1. How did health, health-related behaviour, psychosocial burdens and resources differ at the end of the pandemic between children and adolescents from families at risk of poverty and those with higher incomes?
2. Are these differences independent of the educational status of the parents?

## 2. Methods

### 2.1 Study design and sample

The analyses were carried out using data from the study German Children’s Health Update (KIDA) (see Infobox), which is part of the study German Health Update (GEDA 2022/2023). The GEDA study is a cross-sectional survey conducted by the Robert Koch Institute to assess the health status of individuals aged 16 and older living in private households with their main residence in Germany at the time of data collection [23]. The survey is based on a dual-frame telephone sample, incorporating both mobile and landline phone numbers [24]. Between February 2022 and April 2023, the study design of GEDA was expanded to invite participating parents of children and adolescents aged 3 to 15, as well as 16- and 17-year-old adolescents themselves to take part in the KIDA study [23]. The KIDA study was designed to examine the health status and health-related behaviour of children and adolescents during the COVID-19 pandemic. Initially, telephone interviews were conducted with parents of 3- to 15-year-old children and with 16- to 17-year-old adolescents. Subsequently, between April 2022 and June 2023, a follow-up online survey was conducted with the same respondents.

For this analysis, only parental reports on children and adolescents aged 3 to 15 years were included. The analyses are based on data from 6,514 participants from the telephone survey and 2,760 participants from the online survey.

### 2.2 Indicators

The definition of risk of poverty in Germany and other member states of the European Union (EU) follows a relative concept of poverty. According to this, people are considered to be ‘at risk of poverty’ if they live in a household with such a low income that the household members are denied what is considered a ‘normal’ standard of living [6, 8]. According to the official definition, the risk-of-poverty threshold is undercut if the equivalised disposable income is less than 60 % of the median income of the total population [8, 25]. In this analysis based on KIDA data, the income groups were categorised using the median equivalised disposable income of € 1,982 for 2022 according to the Federal Statistical Office’s microcensus disposable [26] into ‘low’ (< 60 % of the median = at risk of poverty), ‘medium’ (60 % – <150 % of the median) and ‘high’ (≥ 150 % of the median) [25]. In order to calculate the equivalised disposable income, the disposable household income was divided by the sum of the means weights of all household members according to the new OECD scale [27]. This scale assigns a needs weighting of 1.0 to the first adult in the household, a needs weighting of 0.5 to each additional person in the household and a needs weighting of 0.3 to household members under the age of 14. The needs weighting is used to take cost savings in multi-person households through joint economic activity into account when determining the financial situation or risk of poverty of a household. Missing values for the equivalised disposable income were imputed using a multiple regression model. For this purpose, the respondents‘ information on their age, education and employment status as well as regional statistical information on the average disposable household income of the respondents’ region of residence were used. Further socio-demographic characteristics considered were the age of the children and adolescents (3 – 10 years and 11 – 15 years), the gender recorded on the birth certificate (female, male; children with an open gender entry were not taken into account) and the household’s highest level of education. The highest educational qualification of the household included the school and vocational qualifications of both parents. Using the CASMIN classification (Comparative Analyses of Social Mobility in Industrial Nations), the educational qualifications were categorised as ‘low’, ‘medium’ and ‘high’ [28].

**KIDA – German Children’s Health Update Monitoring child health during and after the COVID-19 pandemic**

**Data holder:** Robert Koch Institute

**Objectives:** provision of reliable information on the physical and mental health status and health-related behaviour of children and adolescents aged 3 to 17 years.

**Study design:** nationwide cross-sectional telephone survey and follow-up in-depth online survey.

**Population:** parents of children aged 3 – 15 years and adolescents aged 16 – 17 years who are integrated in the ongoing GEDA study.

**Sampling:** random sample of landline and mobile phone numbers (dual frame method) from the sampling system of the ADM (Arbeitskreis Deutscher Markt- und Sozialforschungsinstitute e.V.).

**Sample size:** telephone sample = 6,992 participants, online sample = 2,894 participants

**Study period:** February 2022 until June 2023

Further information in German is available at

www.rki.de/kida

The following outcome variables were selected to represent different dimensions of health (see Annex Table 1):

► Health status: parent-rated general health, parent-rated general mental health, increased care or support need, obesity
► Health-related behaviour: participation in voluntary sports activities at school, participation in organised sports clubs or commercial sports activities during leisure time, utilisation of psychosocial support services
► Psychosocial burdens and resources: support in the educational and private environment, burdens due to financial constraints, cramped living conditions, and conflicts in the family

### 2.3 Statistical analyses

In the first step, stratified prevalences by income group, 95 % confidence intervals and *p*-values by chi-square were calculated separately for the overall group and by gender. For the health indicators from the telephone survey, Poisson regression was also used to check whether the interaction between gender and income is significant. Due to the small sample size, only the values for the prevalences of the overall group are reported for the health indicators from the online survey; results stratified by gender are only shown graphically. If the lower limit of the confidence interval is less than 2/3 of the prevalence, the relevant result is marked as statistically uncertain in the figure (*).

In the second step, Poisson regression was used to calculate adjusted prevalence ratios with 95 % confidence intervals for each health outcome. The prevalence ratio indicates the ratio of the prevalences of a group and a reference group. In Model 1, the results were adjusted for gender and age. In Model 2, the highest parental educational qualification of the household was also included in the modelling. Only cases with complete information on age, gender and parental education were included. *P*-values less than 0.05 were considered statistically significant.

All calculations were carried out using a design weighting, which is determined by the selection probability of the individual respondents, and an adjustment weighting, which adapts the sample to the population distribution with regard to the variables region, age, gender and level of education. As not all participants in the KIDA telephone survey also took part in the online survey, drop-out weights were calculated for these respondents using the information from the telephone survey in order to minimise selection effects [23].

## 3. Results

The description of the sample (Annex Table 2) shows that the proportion of children and adolescents at risk of poverty is 16.9 % in the telephone sample of the KIDA study and 11.0 % in the online sample. There is a strong correlation between disposable household income and parental education: 39.0 % of parents from families at risk of poverty have a low level of education, while this applies to only 5.7 % of parents in the high-income group. Similarly, 11.7 % of parents from families at risk of poverty have a high level of education compared to 66.2 % of high-income families.

### 3.1 Results of the bivariate analyses

#### Health status

**Figure 1:**
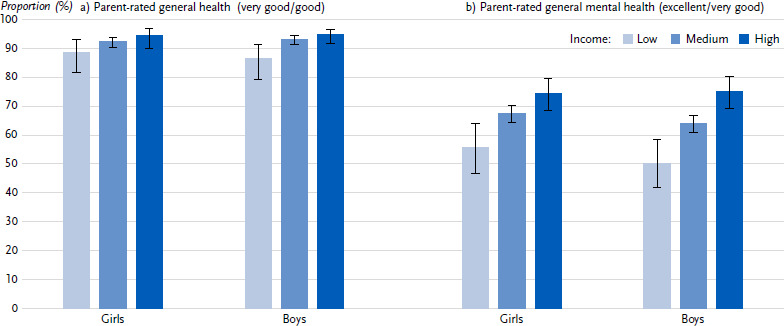

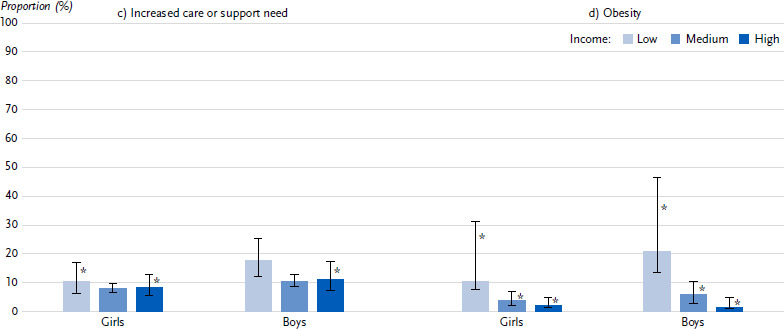
Health of girls and boys stratified by income (parental information; prevalences in %, 95 % confidence intervals). Source: KIDA Study a) Parent-rated general health (*n* = 3,122 girls, *n* = 3,387 boys) b) Parent-rated general mental health (*n* = 3,119 girls, *n* = 3,385 boys) c) Increased care or support need (*n* = 3,100 girls, *n* = 3,360 boys) d) Obesity (*n* = 1, 169 girls, *n* = 1,292 boys) *Due to the small number of cases, the values have a high degree of statistical uncertainty and must therefore be interpreted with caution.

With regard to health status, most indicators differ according to household income to the disadvantage of children and adolescents from families at risk of poverty (Annex Table 3). Their parents are less likely to rate their children’s general health as very good or good (87.4 %) than parents from medium- or high-income families (92.8 % and 94.6 % respectively). Furthermore, children and adolescents from families at risk of poverty are less likely to have excellent or very good parent-rated mental health (52.7 %) than their peers from high-income families (74.8 %). In addition, their parents report an increased care or support need more frequently (14.6 %) than parents with middle- or high-income (9.4 % and 9.8 % respectively). The obesity prevalence is more than three times higher among children and adolescents at risk of poverty (16.5 %) than among their peers from middle-income (5.0 %) or high-income (1.9 %) families. The differences in health according to income groups appear to be slightly more pronounced for boys than for girls, even if the interaction term of income and gender is not statistically significant (based only on the indicators of the telephone survey) (Figure 1).

#### Health-related behaviour

**Figure 2:**
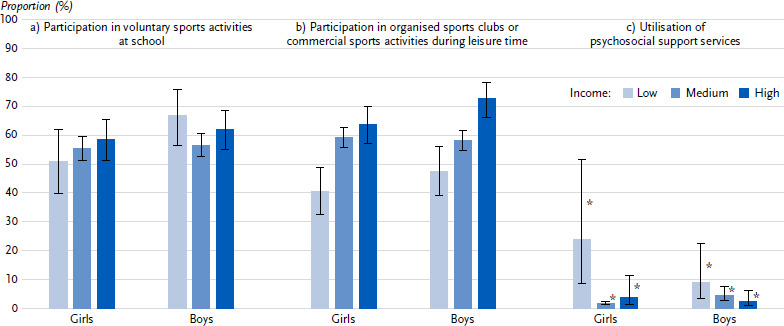
Health-related behaviour of girls and boys stratified by income (parental information; prevalences in %, 95 % confidence intervals). Source: KIDA Study a) Participation in voluntary sports activities at school (*n* = 2,112 girls, *n* = 2,284 boys) b) Participation in organised sports clubs or commercial sports activities during leisure time (*n* = 2,912 girls, *n* = 3,175 boys) c) Utilisation of psychosocial support services (*n =* 1,293 girls, *n =* 1,389 boys) *Due to the small number of cases, the values have a high degree of statistical uncertainty and must therefore be interpreted with caution.

With regard to physical activity behaviour in childhood and adolescence, 57.6 % of parents stated that their child takes part in voluntary sports activities at school (Annex Table 3). Here, no differences were found between income groups. However, there is a statistically significant income gradient when it comes to participation in organised sports clubs or commercial sports activities during leisure time: while 67.9 % of children and adolescents in the high-income group make use of sports clubs or commercial sports activities, 58.7 % in the middle-income group and 44.5 % in families at risk of poverty do so. For both indicators, there were no differences between girls and boys in terms of the association with income (Figure 2). Psychosocial support services to overcome challenges in connection with the COVID-19 pandemic were utilised more frequently for or with children and adolescents at risk of poverty by their parents (15.9 %) than for children and adolescents in the middle- and high-income groups (3.1 % each) (Annex Table 3).[Fig fig003]

#### Psychosocial burdens and resources

At the end of the pandemic, 52.3 % of children and adolescents from families at risk of poverty were burdened by financial restrictions, while this applied to 23.5 % of their peers from families in the middle-income group and 13.3 % from families in the high-income group (Annex Table 3). Burdens due to cramped living conditions were reported for just under a quarter of children and young people from families at risk of poverty. In families with a medium or high income, these burdens are lower (13.1 % and 10.5 % respectively). There are no statistically significant differences for stress caused by conflicts within the family.

**Figure 4:**
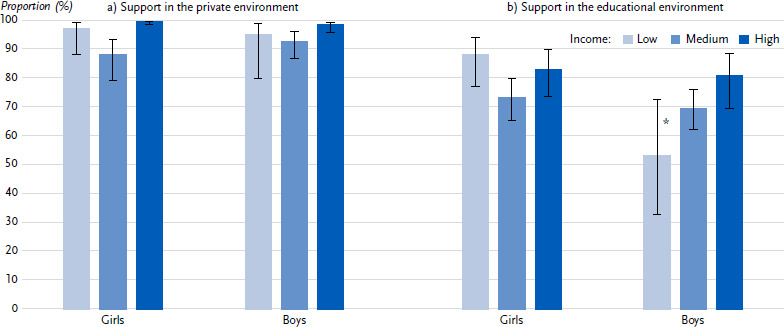
Psychosocial resources of girls and boys stratified by income (parental information; prevalences in %, 95 % confidence intervals). Source: KIDA Study a) Support in the private environment (*n* = 965 girls, *n =* 1,041 boys) b) Support in the educational environment (*n* = 964 girls, *n =* 1,038 boys) *Due to the small number of cases, the values have a high degree of statistical uncertainty and must therefore be interpreted with caution.

In addition, children and adolescents from middle-income families (90.2 %) are less likely to receive private support than their peers from high-income families (98.8 %) (Annex Table 3). The proportion of children and young adolescents at risk of poverty who receive support in the private environment (95.7 %) lies between the middle- and high-income groups. In the school environment, 67.2 % of children and young people from low-income families, 71.3 % from middle-income families and 81.7 % from high-income families receive support, although the differences are not statistically significant. Gender-stratified analysis shows, however, that girls at risk of poverty receive support in the school environment significantly more often than boys at risk of poverty (Figure 4).

### 3.2 Results of the multivariate analyses

Controlling for gender and age (Model 1) in the multivariate regression analyses, the bivariate-observed differences by income remain (Table 1). Exceptions are the increased need for care and support, and support in the private environment, where there are no longer any statistically significant differences in income.

When parents’ level of education is added to the modelling (Model 2), the higher prevalence ratios for children and adolescents at risk of poverty are partially reduced compared to Model 1, but remain statistically significant. This applies to parents’ assessments of their children’s mental health, obesity, participation in sports clubs or commercial sports activities, use of psychosocial services and the burden of financial constraints. Only in the case of general health are there no longer any statistically significant differences in income after additionally controlling for parents’ level of education.

## 4. Discussion

The KIDA study describes the health status of children and adolescents at the end of the COVID-19 pandemic in Germany. The results show that children and adolescents at risk of poverty had poorer health opportunities in 2022/2023 than their peers from financially better-off families: they have poorer parent-rated general and mental health, are more likely to have increased care and support needs and to be obese; they are more often burdened by financial worries and cramped living conditions and are more likely to make use of psychosocial support services with their parents. In addition, they receive less support in their private environment and are less likely to take part in sports clubs or commercial sports activities during leisure time, while they participate in voluntary sports activities at school at a similar rate to their peers in higher income groups.

The reported results are largely in line with existing research findings: living in conditions with a risk of poverty and the experience of deprivation, shame and marginalisation that often go hand in hand with this are associated with impaired mental health in children and adolescents in particular [6]. This is already evident in previous nationwide studies, such as the German Health Interview and Examination Survey for Children and Adolescents (KiGGS Wave 2) from 2014 to 2017 [2]. Here, however, emotional and behavioural problems were measured as indicators of mental health (Strengths and Difficulties Questionnaire, SDQ), so that the results are not directly comparable. Data from the Scientific Institute of the AOK for the pandemic period also show that low-income mothers are significantly less likely to rate their children’s mental health as very good or good, and more likely to report a deterioration in their children’s mental health during the pandemic than mothers with a medium or high disposable household income [22].

With regard to the general health of their children as assessed by their parents, KiGGS Wave 2 (2014-2017) also shows that children and adolescents at risk of poverty are in poorer health than their peers from families with higher incomes [2]. However, while, in KiGGS Wave 2, 92.0 % of children and adolescents at risk of poverty were assessed by their parents as being in very good or good health [2], the KIDA study found this percentage to be only 87.4 %. Nevertheless, lower prevalences were also found for the middle- and high-income groups in the KIDA study than in KiGGS Wave 2. This may be due to a generally poorer assessment of health during the pandemic. However, it should be noted that the prevalences are not directly comparable due to the differences in the age range (KiGGS Wave 2: 3 – 17 years), the sampling design and the survey methods.

The increased care or support need observed here among children and adolescents at risk of poverty were measured using an item from the Children-with-Special-Health-Care-Needs-Screeners (CSHCN) and are therefore not comparable with results based on the overall screener or other items from the screener [2, 29]. During the COVID-19 pandemic, results from a study in Germany show that children with higher care needs (measured with the CSHCN Total Screener) receive less social support if they live in a family with a low socio-economic status than their peers from more affluent families [29]. No studies can be found specifically on the care and support needs of children and adolescents at risk of poverty after the pandemic, showing a research gap for Germany.

The higher prevalence of obesity observed among children and adolescents at risk of poverty is consistent with a nationwide study conducted before the pandemic [2]. The income-related differences in obesity prevalence appear more pronounced in the KIDA study compared to KiGGS Wave 2. However, in KiGGS Wave 2, body height and weight were obtained using standardized procedures in examination centres. In the KIDA study, these data are based on parental reports, which are less valid than objectively measured values [30]. It is evident that parents are often unable to provide accurate information on body height and weight, especially for older children and adolescents. In the KIDA study, the data were additionally collected via an online survey, which involved a smaller sample size, thereby resulting in prevalence estimates with greater statistical uncertainty. The association between poverty and obesity is mediated, among other factors, by diet and exercise: poverty can impede access to healthy food options [10], and to opportunities for sports and physical exercise [31]. The findings of the KIDA study also indicate this for participation in sport activities during leisure time.

According to the KIDA data, children and adolescents in families at risk of poverty are less likely to participate in sports clubs or commercial sports activities during leisure time, which is comparable with the results from KiGGS Wave 2 on sporting activity during leisure time in general [2]. Unlike in KiGGS Wave 2, however, the KIDA study explicitly asked whether they participate in sports clubs or commercial sports activities, i.e. organised sports activities. According to analyses of the Socio-Economic Panel, adolescents at risk of poverty aged 17 also make use of sports activities in clubs less frequently than adolescents not at risk of poverty [32]. Many commercial sports facilities (such as gyms, tennis lessons, ballet or swimming schools) involve costs that are a major barrier for families at risk of poverty [32, 33]. Support benefits from the education and participation package, a state support programme for young people from low-income families (e.g. subsidies for sports club fees), are often not claimed due to the perceived stigmatisation and shame involved. In addition, lack of awareness of the entitlement to benefits as well as the complicated application process often lead to non-utilisation of the support services [34]. It can also be assumed that high-priced commercial sports programmes are offered less frequently in socio-economically deprived residential areas. There are also associations between the physical activity behaviour of parents and their children [35]. Factors such as the parents’ sporting activity, their support and attitudes towards sport are discussed in this context as important factors for the child’s sporting and physical activity [36, 37].

In contrast to sports activities during leisure time, KIDA data shows that children and adolescents at risk of poverty participate in voluntary sports activities at school at a similar rate to their peers from families with higher incomes. Before the pandemic, there was even evidence of more frequent participation in voluntary sports activities at school among 17-year-old adolescents from households at risk of poverty [32]. Children and adolescents at risk of poverty can therefore be reached effectively with school programmes. The possibility of using these programmes free of charge could have great relevance here [32].

In addition to the increased need for care and support among children and adolescents at risk of poverty, the KIDA study also observed a higher utilisation of psychosocial support services by parents and children and adolescents at risk of poverty. However, on the basis of the KIDA study data, it was not possible to answer the question of whether the need for support was met – at least in part – by the services utilised. Analyses included in the KIDA quarterly reports [23], also show that parents at risk of poverty are more often unaware of the support services mentioned in the survey than families with medium or high incomes.

The increased burden on children and adolescents from families at risk of poverty due to cramped living conditions during the pandemic is supported by analyses using data from the Socio-Economic Panel [38]. In these analyses, cramped living space are most frequently reported by families receiving unemployment benefit II. The higher burden of cramped living conditions on children and adolescents at risk of poverty emphasises the importance of sufficient living space for the well-being of young people. The relevance of quantitatively and qualitatively sufficient living space is recognised, for instance, as important for a good family climate, space for socialising with friends and opportunities for retreat [3], which is why ‘sufficient living space’ was also defined as a goal for all member states within the European Child Guarantee [39]. In addition to the burdens caused by cramped living conditions, financial burdens for children and adolescents at risk of poverty were also reported most frequently. In 2022 and 2023 in particular, expenditure on food and heating costs, for example, rose sharply [40]. The limited financial resources and the associated restrictions for families at risk of poverty were therefore also noticeable with the children.

Although, according to the KIDA data, children and adolescents at risk of poverty are more likely to experience psychosocial burdens and health problems and have an increased need for care and support, the results show that, at the end of the pandemic, only two out of three children and adolescents always or mostly received support in the school environment. In the high-income group, this figure is eight out of ten children and adolescents. Even if the differences in income are not statistically significant, there is still a need for action here. With regard to the support perceived by parents for their children in the private environment, there are no differences according to family income. In all income groups, more than nine out of ten children and adolescents receive sufficient support in their private environment. However, in answer to this question, parents themselves rated the support they provide; it can be assumed that an assessment by the young people themselves could be somewhat different. Nevertheless, good support from the private environment is an important resource, especially for children and adolescents at risk of poverty.

The question of whether and to what extent income differences in health, health-related behaviour and psychosocial burdens and resources differ between girls and boys is not analysed in depth in this article. This would require studies with a larger sample size. The sample size is too small for a simultaneous stratification by income and gender, especially for the indicators collected in the online survey. Existing differences could therefore be underestimated or overlooked (ß-error). The results presented here in graphical form should therefore be viewed with great caution.

Even if the differences by gender with regard to the associations between income and health are not statistically significant, the associations appear to be stronger for boys than for girls. With regard to sports activities, it is known that boys participate more frequently than girls in organised sports clubs or commercial sports activities in their leisure time [41]. The association between income and participation in sports clubs or commercial sports activities during leisure time and participation in voluntary sports activities at school does not vary statistically significantly by gender. However, in the case of voluntary sports activities at school, differences tend to emerge in the association between participation and income: while girls at risk of poverty are slightly less likely to take part in voluntary sports activities clubs at school than girls in other income groups, boys at risk of poverty are slightly more likely to take part in these programmes than boys in other income groups. Support in the school environment is reported more frequently for girls by parents at risk of poverty than by parents with medium and high incomes. This is less often the case for boys. Whether this corresponds to reality or whether girls at risk of poverty report more frequent support in the school context than boys, or whether parents at risk of poverty are more dissatisfied with the support received by boys cannot be assessed with the data from the KIDA study.

The second research question aims to determine whether the reported differences in health, health-related behaviour and psychosocial burdens and resources can be attributed to a lower level of formal education among parents -which is often associated with poverty. Although some of the analysed associations with income are weakened, they basically hold up when the parents’ level of education is taken into account. This applies to parent-rated mental health, obesity, participation in sports clubs or commercial sports activities, utilisation of psychosocial services and the burden of financial constraints. This indicates an independent relevance of the income situation for these health aspects, which goes beyond the health relevance of education. In this respect, the KIDA results support previous findings for adulthood, which are available both internationally and for Germany and confirm the independent relevance of poverty or financial disadvantage as a social determinant of health [42, 43]. Only in the case of parent-rated general health are there no longer any statistically significant differences in income after adjustment for parents’ level of education.

### 4.1 Strengths and limitations

The strength of the KIDA study is that it provides data on a large number of health-related, social and socio-economic indicators as well as for different age groups in childhood and adolescence at the end of the pandemic, which also enables conclusions to be drawn regarding the health status of children and adolescents at risk of poverty.

One limitation of the present analysis is that the KIDA study is only partially representative of the nationwide population of children and adolescents. Access via the GEDA study resulted in no direct random selection of children and adolescents but of one parent. In addition, only some of the parents from the GEDA study took part in the KIDA study. Then again, fewer parents took part in the online survey than in the telephone survey. This dropout rate may have led to a selection of participants. For example, it cannot be ruled out that parents with high psychosocial burdens participated less frequently in the KIDA study. In particular, parents with a low level of education were less likely to take part in the KIDA study. A complex weighting procedure was used in order to be able to make population-based conclusions.

The combination of telephone and online survey was also accompanied by a change in the survey mode, which may have contributed to different response behaviour. When interpreting the results, it should also be considered that the data for 3- to 15-year-olds is based on information provided by their parents, which may differ from the children’s and adolescents’ self-reporting, particularly with regard to information on mental health, height or body weight [44]. As the KIDA study is a cross-sectional survey, no conclusions can be drawn from the analyses regarding causality or direction of effect.

### 4.2 Conclusions

At the end of the pandemic, the health of children and adolescents at risk of poverty continues to be appreciably worse than that of their peers from less precarious living conditions. Even though there is no clear evidence of an increase in health inequalities in childhood and adolescence in Germany at the end of the pandemic [12, 19–21], it can be assumed that children and adolescents growing up in poor living conditions will be more affected in the long term in terms of their well-being and their ability to cope with developmental tasks by the burdens of the pandemic, but also by current crises such as climate change, military conflicts within and outside Europe, and the economic downturn. Important developmental steps and experience in childhood and adolescence that were missed out during the pandemic or due to a lack of financial or social resources may not be recoverable, or only to a limited extent [45]. It is therefore possible that the increase in health inequalities in this cohort will only appear with a time lag, which is why future studies should pay particular attention to it and continue to monitor it from the perspective of health equity.

It is therefore particularly important to increase activities that improve both the living conditions and the individual health of children and adolescents living in poverty. While designing these interventions, the specific family circumstances of children and adolescents at risk of poverty should be taken into account; for example, if they live in single-parent households or families with many children. Health promotion is to be understood as a cross-sectional task that, in line with the World Health Organisation’s (WHO) health-in-all-policies approach, requires measures in various policy areas and thus the cooperation of many political departments as well as actors with practical experience and those from science and civil society [46]. Strategies to combat child and family poverty and to reduce disadvantages and marginalisation of children and adolescents living in poverty and at risk of poverty are at the top of the agenda. To this purpose, the recommendation on the introduction of a European Child Guarantee was adopted at European level in 2021 [39], which complements the European Union’s comprehensive children’s rights strategy [47]. This strategy is being implemented in Germany through the National Action Plan ‘New Opportunities for Children’ (‘Neue Chancen für Kinder’) [48], which was adopted in 2023 and aims to ensure better access for socially disadvantaged children and adolescents to early childhood care, education and upbringing, school-related activities, at least one healthy meal per school day, healthy food and adequate housing as well as healthcare. In the first progress report on the implementation of the National Action Plan for 2025, a statement from different civil society actors calls, among other things, for the formulation of measurable and time-bound targets that will allow monitoring the progress in reducing child poverty [49].

In addition to these infrastructural improvements, the introduction of a basic child protection guarantee is called for to ensure that all children and adolescents have a minimum standard of living, to protect them from poverty and to improve their opportunities for participation [50]. The UN Convention on the Rights of the Child recognises ‘[…] the right of every child to a standard of living adequate for the child’s physical, mental, spiritual, moral and social development’ [1]. The National Action Plan ‘New Opportunities for Children’ therefore also aims to increase parental labour-force participation, which can contribute to families’ economic stability [48]. The Children’s Rights Network is calling for all measures taken by the Federal Government to be scrutinised in terms of how they affect the situation of children and adolescents, and, in particular, groups at risk of poverty [51].

In order to adequately address the complexity of promoting health and reducing health inequalities in childhood and adolescence, political strategies and measures at federal and state levels need to be complemented by measures at the level of local communities and local living environments. According to the interministerial working group ‘Health effects of the coronavirus on children and adolescents’, measures to promote the health of children and adolescents should be connected to regulatory structures such as daycare centres or schools, which are accessible to all young people at a low threshold and without stigmatisation [52]. This recommendation is supported by the results of the KIDA study presented here, which show that children and adolescents at risk of poverty can be reached just as well as their peers from more affluent backgrounds through sports activities in a school context. Prevention chains are recognised as a suitable structural approach to prevention in order to coordinate the various measures and services offered by a local authority to promote health and reduce health inequalities in a targeted manner [53]. Prevention chains are designed to establish an integrated overall strategy and a sustainable network of support, counselling and promotion at a local level and with the participation of children, adolescents and families [53].

Beside this bundle of measures, Germany needs a nationwide and continuous monitoring of health and health inequalities in childhood and adolescence in order to recognise long-term trends, and to be able to rapidly identify problems in acute crises such as a pandemic. Furthermore, evaluation studies are needed to provide information on which measures are effective in reducing child poverty and health inequalities in practice.

## Figures and Tables

**Figure 3: fig003:**
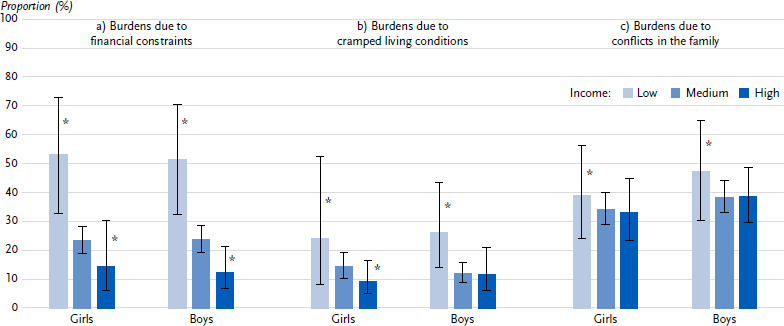
Psychosocial burdens of girls and boys stratified by income (parental information; prevalences in %, 95 % confidence intervals). Source: KIDA Study a) Financial constraints (*n =* 1,285 girls, *n =* 1,376 boys) b) Cramped living conditions (*n =* 1,285 girls, *n =* 1,372 boys) c) Conflicts in the family (*n =* 1,284 girls, *n =* 1,376 boys) *Due to the small number of cases, the values have a high degree of statistical uncertainty and must therefore be interpreted with caution.

**Table 1:** Health status, health-related behaviour and psychosocial burdens and resources of children and adolescents at risk of poverty compared to their peers from middle- and high-income families (prevalence ratios, 95 % confidence intervals, *p*-values). Source: KIDA Study

		Model 1 (adjusted for age and gender)			Model 2 (adjusted for age, gender and education)		
	Income	PR	(95 % CI)	*p*-values	PR	(95 % CI)	*p*-values
**Parent-rated general health** *n =* 6,500	Low		Ref.			Ref.	
	Medium	1.06	(1.01 – 1.12)	0.029	1.04	(0.98 – 1.09)	0.183
	High	1.08	(1.02 – 1.15)	0.008	1.04	(0.98 – 1.10)	0.194
**Parent-rated general mental health** *n =* 6,496	Low		Ref.			Ref.	
	Medium	1.24	(1.10 – 1.41)	< 0.001	1.20	(1.06 – 1.36)	0.003
	High	1.41	(1.24 – 1.60)	< 0.001	1.30	(1.14 – 1.48)	<0.001
**Increased care or support need** *n =* 6,452	Low		Ref.			Ref.	
	Medium	0.64	(0.45 – 0.93)	0.019	0.77	(0.53 – 1.13)	0.182
	High	0.68	(0.43 – 1.09)	0.108	0.93	(0.55 – 1.55)	0.773
**Obesity** *n* = 2,459	Low		Ref.			Ref.	
	Medium	0.31	(0.12 – 0.78)	0.013	0.40	(0.18 – 0.91)	0.029
	High	0.12	(0.04 – 0.34)	< 0.001	0.19	(0.07 – 0.49)	0.001
**Participation in voluntary sports activities at school** *n* = 4,388	Low		Ref.			Ref.	
	Medium	0.94	(0.82 – 1.09)	0.418	0.97	(0.83 – 1.12)	0.637
	High	1.00	(0.86 – 1.17)	0.978	1.03	(0.88 – 1.22)	0.696
**Participation in organised sports clubs or commercial sports activities during leisure time** *n =* 6,078	Low		Ref.			Ref.	
	Medium	1.31	(1.12 – 1.53)	0.001	1.17	(1.00 – 1.36)	0.050
	High	1.52	(1.29 – 1.79)	< 0.001	1.26	(1.07 – 1.48)	0.006
**Utilisation of psychosocial support services** *n* = 2,680	Low		Ref.			Ref.	
	Medium	0.20	(0.08 – 0.50)	0.001	0.22	(0.09 – 0.52)	0.001
	High	0.20	(0.07 – 0.58)	0.003	0.20	(0.07 – 0.60)	0.004
**Support in the private environment** *n* = 2,004	Low		Ref.			Ref.	
	Medium	0.94	(0.88 – 1.01)	0.078	0.91	(0.84 – 1.00)	0.042
	High	1.03	(0.98 – 1.08)	0.313	0.97	(0.90 – 1.04)	0.394
**Support in the educational environment** *n* = 2,000	Low		Ref.			Ref.	
	Medium	1.04	(0.82 – 1.33)	0.740	1.01	(0.80 – 1.26)	0.960
	High	1.21	(0.94 – 1.54)	0.134	1.11	(0.88 – 1.41)	0.364
**Burdens due to financial constraints** *n* = 2,659	Low		Ref.			Ref.	
	Medium	0.45	(0.32 – 0.64)	< 0.001	0.49	(0.35 – 0.69)	<0.001
	High	0.26	(0.14 – 0.46)	< 0.001	0.31	(0.16 – 0.59)	<0.001
**Burdens due to cramped living conditions** *n* = 2,655	Low		Ref.		Ref.	Ref.	
	Medium	0.53	(0.29 – 0.97)	0.040	0.55	(0.31 – 0.96)	0.037
	High	0.43	(0.21 – 0.88)	0.021	0.43	(0.21 – 0.89)	0.024
**Burdens due to conflicts in the family** *n* = 2,658	Low		Ref.			Ref.	
	Medium	0.85	(0.61 – 1.18)	0.336	0.81	(0.57 – 1.15)	0.243
	High	0.84	(0.58 – 1.22)	0.359	0.74	(0.49 – 1.11)	0.144

PR = Prevalence Ratio, 95- % CI = 95 %-Confidence interval, Ref. = Reference category

*Reading example:* A PR of 1.41 for the high-income group means, for example, that the prevalence of excellent or very good mental health is 1.41 times higher among children and adolescents from high-income households than among those at risk of poverty (Model 1).

## Appendix

**Annex Table 1:** Description of the outcome variables on health, health-related behaviour and psychosocial burdens and resources

Variable (T = Telephone/O = Online)	Instrument [Source]	Question(s)	Categories
**Health status**			
Parent-rated general health (T)	[54, 55]	‘How would you describe your child’s state of health in general?’	1 = ‘very good’, ‘good’; 0 = ‘moderate’, ‘bad’, ‘very bad’
Parent-rated general mental health (T)	[56]	‘How would you rate your child’s mental health in general?’	1 = ‘excellent’, ‘very good’, 0 = ‘good’, ‘not so good’, ‘poor’
Increased care or support need (T)	‘Children with Special Health Care Needs (CSHCN)’-Screener: contains a single item from the German translation of the short questionnaire [57, 58]	‘Does your child need more medical care, psychosocial or educational support than usual for children of the same age?’ (filter question) If yes: 1) ‘Is this due to an illness, behavioural disorder or a health problem?’ 2) ‘Has this problem lasted 12 months or is it expected to last longer than 12 months?’	1 = ‘yes’, 0 = ‘no’
Obesity (O)	Body weight and height; obesity is defined by the Kromeyer-Hauschild et al reference system as a body mass index (BMI) above the 97th percentile, taking into account age and gender [59, 60]	‘How much does your child weigh without clothing? The weight can be given to one decimal place.’ ‘How tall is your child? Height in cm.’	1 = obesity (>97th percentile), 0 = no obesity (≤ 97th percentile)
Participation in voluntary exercise or sports activities at school (T)	Based on schools in regular operation	‘Now please think about the last 4 weeks: Has your child participated in physical education or sports clubs at school?’	1 = ‘yes’, 0 = ‘no’
Participation in organised sports clubs or commercial sports activities during leisure time (T)		‘Now please think about the last 4 weeks: Has your child taken part in sports club programmes or sports courses in fitness studios, ballet or swimming lessons, etc.?’	1 = ‘yes’, 0 = ‘no’
Utilisation of psychosocial support services (O)	Utilisation of psychosocial support services to deal with the challenges associated with the COVID-19 pandemic	‘Which of the following support services have you utilised with or for your child in the last four weeks in order to cope better with the challenges associated with the COVID-19 pandemic?’ 1) Local psychosocial support and counselling services (e.g. family or parent-child counselling centres, self-help groups, parent-child café meetings, etc.) 2) Online portals with information and tips on psychosocial health, such as ‘psychisch stabil bleiben (staying mentally stable)’, ‘Corona und du (Coronna and you’), ‘angstfrei.news’, or the family portal 3) Online training to strengthen psychosocial health, such as ‘get.calm and move.on’, ‘stark durch die Krise (strong through the crisis)’ 4) Health apps to strengthen mental health, such as ‘iFightDepression’ or the ‘Krisenkomass (CrisisCompass) App’ 5) Telephone-based psychosocial support and counselling, such as the ‘Nummer gegen Kummer (Number Against Worries)’ helpline	1 = ‘yes’, 0 = ‘no, no need’, ‘no, service is not known’, ‘no, other reasons’
Support in the private or educational environment (O)		6- bis 15-year old school children: 1) ‘Help/support from teachers or other people in the school or workplace environment’ 2) ’Help/support from a parent or other person in the private environment’	1 = ‘mostly’, ‘always’, 0 = ‘never’, ‘rarely’, ‘sometimes’
Burdens due to financial constraints, cramped living conditions or conflicts in the family (O)		‘To what extent has your child felt burdened by the following events/incidents in the last four weeks?’	1 = ‘very highly burdened’ to ‘slightly burdened’, 0 = ‘not burdened’, ‘does not apply’

**Annex Table 2:** Sample description. Source: KIDA Study

		Telephone		Online	
	Age (in years)	*n* unweighted	% weighted	*n* unweighted	% weighted
**Total**	**3 – 15**	**6,514**		**2,760**	
**Gender**	3 – 15				
Female		3,122	48.59	1,333	49.88
Male		3,389	51.39	1,427	50.12
Divers		3	0.02	0	
Missing		0		0	
**Age**	3 – 15				
3 – 10 years		3,658	61.81	1,565	63.84
11 – 15 years		2,856	38.19	1,195	36.16
**Income**	3 – 15				
Low		569	16.85	173	10.97
Medium		4,499	69.59	1,948	75.72
High		1,446	13.57	639	13.32
Missing		0		0	
**Parents’ level of education**	3 – 15				
Low		193	16.61	63	13.34
Medium		2,038	50.15	800	48.81
High		4,274	33.23	1,895	37.85
Missing		9		2	
**Health**					
**Parent-rated general health**	3 – 15				
Very good/Good		6,138	92.25		
Moderate/poor/very poor		374	7.75		
Missing		2			
**Parent-rated general mental health**	3 – 15				
Excellent/very good		4,465	65.11		
Good/not so good/poor		2,042	34.89		
Missing		7			
**Increased care or support need**	3 – 15				
Yes		572	10.24		
No		5,891	89.76		
Missing		51	0		
**Obesity**	3 – 15				
Yes				88	4.93
No				2,373	95.07
Missing				299	
**Health-related behaviour**					
**Participation in voluntary exercise or sports activities clubs at school**	5 – 15				
Yes		2,463	57.41		
No		1,935	42.59		
Missing		572			
**Participation in organised sports clubs or commercial sports activities during leisure time**	3 – 15				
Yes		3,977	57.59		
No		2,113	42.41		
Missing		424			
**Utilisation of psychosocial support services**	3 – 15				
Yes				118	6.06
No				2,564	93.94
Missing				78	
**Psychosocial burdens and resources**					
**Support In the private environment**	6 – 15				
Mostly/always				1,927	92.96
Sometimes/rarely/never				79	7.04
Missing				134	
**Support In the educational environment**	6 – 15				
Mostly/always				1,540	73.31
Sometimes/rarely/never				462	26.69
Missing				138	
**Burdens due to financial constraints**	3 – 15				
Very highly loaded to slightly loaded				571	28.83
Not burdened/does not apply				2,090	71.17
Missing				99	
**Burdens due to cramped living conditions**	3 – 15				
Very highly burdened to slightly burdened				342	16.39
Not burdened/does not apply				2,315	83.61
Missing				103	
**Burdens due to conflicts in the family**	3 – 15				
Very highly burdened to slightly burdened				1,102	42.41
Not burdened/does not apply				1,558	57.59
Missing				100	

**Annex Table 3:** Health, health-related behaviour and psychosocial burdens and resources of children and adolescents by income group (weighted prevalences)* (Telephone n = 6,514; Online *n* = 2,760). Source: KIDA Study

Indicator	Total						Girls						Boys					
	Family income						Family income						Family income					
	Low (<60 %)		Medium (60 % – <150 %)		High (>150 %)		Low (<60 %)		Medium (60 % – <150 %)		High (>150 %)		Low (<60 %)		Medium (60 % – <150 %)		High (>150 %)	
	%	(95 % CI)	%	(95 % CI)	%	(95 % CI)	%	(95 % CI)	%	(95 % – KI)	%	(95 % CI)	%	(95 % CI)	%	(95 % CI)	%	(95 % CI)
Parent-rated general health (Very good/good)	87.4	(82.1 – 91.4)	92.8	(91.4 – 94.0)	94.6	(91.8 – 96.4)	88.7	(81.6 – 93.3)	92.3	(90.2 – 94.0)	94.4	(89.9 – 97.0)	86.4	(78.9 – 91.5)	93.2	(91.2 – 94.8)	94.7	(91.6 – 96.7)
Parent-rated general mental health (Excellent/very good)	52.7	(46.4 – 58.8)	65.6	(63.3 – 67.8)	74.8	(70.5 – 78.6)	55.7	(46.7 – 64.3)	67.4	(64.1 – 70.5)	74.5	(68.4 – 79.8)	50.2	(41.6 – 58.8)	63.9	(60.8 – 67.0)	75.1	(69.0 – 80.3)
Increased care or support need	14.6	(10.4 – 20.2)	9.4	(8.1 – 10.9)	9.8	(7.0 – 13.4)	10.6	(6.3 – 17.4)	8.0	(6.4 – 9.9)	8.5	(5.5 – 13.0)	17.8	(12.0 – 25.7)	10.7	(8.8 – 13.1)	11.3	(7.1 – 17.5)
Obesity	16.5	(7.2 – 33.6)	5.0	(3.0 – 8.3)	1.9	(1.0 – 3.9)		**–**		**–**		**–**		**–**		**–**		**–**
Participation in voluntary exercise or sports activities clubs at school	59.8	(51.9 – 67.2)	56.0	(52.8 – 59.1)	60.1	(54.7 – 65.2)	51.1	(39.9 – 62.3)	55.4	(51.1 – 59.6)	58.4	(50.9 – 65.5)	66.8	(56.4 – 75.9)	56.6	(52.3 – 60.8)	61.9	(54.7 – 68.6)
Participation in organised sports clubs or commercial sports activities during leisure time	44.5	(38.1 – 51.0)	58.7	(56.0 – 61.3)	67.9	(62.9 – 72.5)	40.4	(32.2 – 49.1)	59.3	(55.6 – 62.8)	63.8	(56.8 – 70.3)	47.6	(39.0 – 56.4)	58.2	(54.6 – 61.7)	72.6	(66.0 – 78.3)
Utilisation of psychosocial support services	15.9	(7.1 – 31.8)	3.1	(2.0 – 4.8)	3.1	(1.4 – 6.7)		**–**		**–**		**–**		**–**		**–**		**–**
Support in the private environment	95.7	(87.1 – 98.6)	90.2	(84.6 – 94.0)	98.8	(97.3 – 99.5)		**–**		**–**		**–**		**–**		**–**		**–**
Support in the educational environment	67.2	(49.5 – 81.0)	71.3	(65.5 – 76.5)	81.7	(74.0 – 87.5)		**–**		**–**		**–**		**–**		**–**		**–**
Burdens due to financial constraints	52.3	(37.0 – 67.3)	23.5	(20.1 – 27.3)	13.3	(7.8 – 21.6)		**–**		**–**		**–**		**–**		**–**		**–**
Burdens due to cramped living conditions	25.2	(14.1 – 40.9)	13.1	(10.4 – 16.3)	10.5	(6.6 – 16.5)		**–**		**–**		**–**		**–**		**–**		**–**
Burdens due to conflicts in the family	43.6	(31.1 – 57.0)	36.3	(31.1 – 57.0)	36.1	(29.2 – 43.7)		**–**		**–**		**–**		**–**		**–**		**–**

*n* = Sample, CI = Confidence interval, Income categories according to median income in Germany in 2022

*The gender-stratified prevalences are not shown for the outcomes assessed online, as they are partly based on very small numbers of cases and the statistical uncertainty is therefore very high.
